# Single-agent carboplatin in extensive disease small-cell lung cancer patient with liver failure: a case report within the experience of a single institution

**DOI:** 10.1097/CAD.0000000000001057

**Published:** 2021-03-01

**Authors:** Alessandro Di Federico, Elisa Andrini, Monia Sisi, Giacomo Nuvola, Giuseppe Lamberti, Barbara Lenzi, Elisabetta Nobili, Francesco Gelsomino, Andrea Ardizzoni

**Affiliations:** a Department of Experimental, Diagnostic and Specialty Medicine, University of Bologna, Sant’Orsola-Malpighi University Hospital; b Divisione di Oncologia Medica, IRCCS Azienda Ospedaliero-Universitaria di Bologna, Bologna, Italy

**Keywords:** carboplatin monotherapy, liver failure, poor Eastern Cooperative Oncology Group performance status, prognostic scores, small-cell lung cancer

## Abstract

Until recently, platinum-based chemotherapy has represented the benchmark for the treatment of extensive disease small-cell lung cancer (ED-SCLC). ED-SCLC patients are often diagnosed with poor performance status (PS ≥2) and/or compromised organ functions. In fact, up to 63% of ED-SCLC has extensive liver involvement at diagnosis, which correlates with a poor prognosis. Whether to treat patients with tumor-related organ failure is still debated and the selection of those who could benefit from chemotherapy is crucial. Moreover, severe liver impairment contraindicates the administration of etoposide. Among 74 consecutive ED-SCLC patients followed at our institution from January 2017 to November 2019, three patients received single-agent carboplatin as a first-line treatment due to liver failure. We provide a brief description of a former heavy smoker 70-year-old man who was diagnosed with ED-SCLC and severe liver involvement leading to liver failure. The patient received a first-line treatment with single-agent carboplatin, obtaining a partial response, clinical benefit and the normalization of laboratory test, which documented the complete recovery of liver function. The intent of our work is to highlight the feasibility of single-agent carboplatin in ED-SCLC patients with tumor-related hepatic failure but preserved Eastern Cooperative Oncology Group PS, suggesting that this therapeutic option should not be discouraged a priori. Indeed, the identification of specific tools guiding physicians in the selection of patients who might benefit from the treatment is remarkably needed; meanwhile, the use of available prognostic score (e.g. Manchester score) might be of great value and should be considered in clinical practice.

## Introduction

Small-cell lung cancer (SCLC) is a smoke-related, aggressive form of lung tumor that requires systemic treatment since diagnosis. Until recently, platinum-based chemotherapy has represented the standard first-line treatment for extensive disease SCLC (ED-SCLC) with good Eastern Cooperative Oncology Group (ECOG) performance status (PS) and no comorbidities, yielding to a median overall survival (OS) of approximately 10 months [1]. Among platinum agents, carboplatin demonstrated similar activity and efficacy, but different toxicity profiles when compared to cisplatin.

However, ED-SCLC patients are often diagnosed with poor ECOG PS of ≥2 and compromised organ functions, mainly due to the aggressive nature of SCLC, questioning whether these patients are unfit for a platinum-based doublet chemotherapy regimen [2]. Furthermore, up to 63% of cases at diagnosis have extensive liver involvement that is associated with worse outcome [3]. Because etoposide has mainly hepatic metabolism, liver failure that might derive from liver dissemination contraindicates the use of this chemotherapeutic agent [4]. Differently, carboplatin has a predominantly (70%) renal excretion, with a close correlation between its renal clearance and the glomerular filtration rate (GFR) [5].

Among 74 consecutive ED-SCLC patients followed at our institution from January 2017 to November 2019, 3 patients (4%) received single-agent carboplatin as first-line treatment due to liver failure, defined according to National Cancer Institute Common Terminology Criteria for Adverse Events v4.03 in the presence of grade (G) ≥3 serum liver function tests elevation [alanine aminotransferase, aspartate aminotransferase (AST), total bilirubin and alkaline phosphatase (ALP)]. A brief description of one patient who obtained tumor response with complete normalization of laboratory tests is reported as follows.

## Case description

A 70-year-old man, former heavy smoker, was diagnosed with ED-SCLC of the left lung in June 2019. At the time of presentation, jaundice and hepatomegaly were evident at physical examination. Computed tomography (CT) scan showed a lung primary tumor with thoracic lymph nodes and massive liver dissemination. Laboratory tests demonstrated G4 serum bilirubin increase (14.81 mg/dl), G3 AST elevation (303 U/L, upper limit normal 50), and a serum creatinine of 1.8 mg/dl. ECOG PS at the time of the admission was 1. First-line chemotherapy with single-agent carboplatin area under the curve (AUC) 3.5 was started (total dose 300 mg with serum creatinine 1.7 mg/dl, GFR 61.76 ml/min).

The treatment was well tolerated and laboratory tests gradually improved (serum bilirubin level decreased to G3 and AST level to G1 before second chemotherapy cycle) until normalization. The patient received a total of four cycles of chemotherapy with single-agent carboplatin, at increasing doses (maximum dose AUC 4.5). Post-treatment disease evaluation by CT scan documented partial response on both primary tumor and liver metastases.

Nevertheless, after four treatment cycles, the patient developed bone marrow failure, needing the support of granulocyte colony-stimulating factor and blood transfusions, that precluded the chance to receive further treatment. Bone marrow biopsy demonstrated severe hypocellularity, a suggestive finding for the diagnosis of myelofibrosis. The patient died 150 days from the start of treatment.

## Discussion

In clinical practice, the routinely use of prognostic scores, such as Manchester score system, could be helpful to distinguish patients who are suitable for first-line treatment from those who should receive best supportive care [6]. In ED-SCLC, poor ECOG PS, number of metastatic sites, with particular emphasis on liver involvement, significant alterations of serum lactate dehydrogenase (LDH), ALP, albumin, sodium and hemoglobin are associated with poor prognosis [6,7]. In addition, the evidence of liver failure limits the treatment options. The choice to treat patients with tumor-related organ failure is still debated and the selection of those patients who could benefit from chemotherapy is crucial. In this context, the presence of a poor PS due to disease spread should not be a priori exclusion criterion, because it is commonly accepted that those patients might benefit from chemotherapy [8].

Experience with single-agent carboplatin in SCLC is limited but significant. Response rate is reported to be around 60% in untreated SCLC patients, with a median duration of response of 4.5 months [9]. In a cohort of SCLC patients with poor prognosis, the treatment with single-agent carboplatin led to similar outcomes compared to cyclophosphamide, doxorubicin and vincristine combination, with a response rate of 25% and an OS of 15.9 weeks [10].

Herein, we report the feasibility of carboplatin as single-agent in a patient suffering from ED-SCLC with tumor-related visceral crisis, dominated by liver failure. Yet, we also report that two other ED-SCLC patients with severe liver impairment treated at our institution did not benefit from the same treatment (Table 1). None of the patients had a previous history of liver disease, evidence of viral infection or exposure to hepatotoxins. In this setting, reasons for treatment failure might be either tumor-related, such as chemoresistance and tumor-induced complications, or tumor-independent conditions, such as preexisting comorbidities or older age. In our limited experience, both good ECOG PS and normal sodium levels correlated with a better patient outcome, although the only responder had the highest blood concentrations of LDH and bilirubin (Table 1), suggesting their minor role in predicting patient’s outcome. In fact, as previously mentioned, carboplatin by itself represents a highly active agent and could be useful when patient’s conditions allowed to tolerate the treatment.

**Table 1:** Patients’ characteristics before the administration of the first cycle of single-agent carboplatin. Normal range is reported in brackets

	Response to carboplatin	Sex	Age	Sites of metastasis	ECOG PS	Bilirubin mg/dL (<1,2)	AST U/L (<50)	ALT U/L (<50)	ALP U/L (50–220)	Albumin g/L (35–50)	LDH U/L (80–300)	Hemoglobin g/dL (14–18)	Platelets 10^9^/L (160–370)	Sodium mmol/L (136–145)
Case 1	Yes	Male	70 years	Lymph nodes; liver	1	14,81	303	237	899	30–3	6103	13.4	235	139
Case 2	No	Male	79 years	Lymph nodes; liver; bone; adrenal glands; peritoneum	3	8,77	466	457	216	28–9	1939	12.3	105	146
Case 3		Male	53 years	Lymph nodes; liver	2	4,61	184	106	580	35–4	304	10	422	119

ALP, alkaline phosphatase; ALT, alanine transaminase; AST, aspartate transaminase; ECOG PS, Eastern Cooperative Oncology Group Performance Status LDH, lactate dehydrogenase.

Our experience might suggest that the presence of visceral crisis should not discourage the administration of carboplatin as single-agent treatment in selected patients with preserved PS. However, the identification of specific tools being able to guide oncologists in the treatment decision making (in particular, best supportive care versus chemotherapy) in this complex and critical setting is urgently needed.

## Acknowledgements

**Conflicts of interest**

There are no conflicts of interest.
